# Characterization of affective lability across subgroups of psychosis spectrum disorders

**DOI:** 10.1186/s40345-021-00238-0

**Published:** 2021-11-04

**Authors:** Margrethe Collier Høegh, Ingrid Melle, Sofie R. Aminoff, Beathe Haatveit, Stine Holmstul Olsen, Idun B. Huflåtten, Torill Ueland, Trine Vik Lagerberg

**Affiliations:** 1 CoE NORMENT, Norwegian Centre for Mental Disorders Research, Division of Mental Health and Addiction, University of Oslo and Oslo University Hospital, Building 49, Ullevål sykehus, Nydalen, PO Box 4956, 0424 Oslo, Norway; 2 Division of Mental Health and Addiction, Oslo University Hospital, Oslo, Norway; 3 Department of Psychology, University of Oslo, Oslo, Norway

**Keywords:** Affective lability, Bipolar disorders, Schizophrenia, Psychosis spectrum, The Affective Lability Scale-Short Form (ALS-SF)

## Abstract

**Background:**

Affective lability is elevated and associated with increased clinical burden in psychosis spectrum disorders. The extent to which the level, structure and dispersion of affective lability varies between the specific disorders included in the psychosis spectrum is however unclear. To have potential value as a treatment target, further characterization of affective lability in these populations is necessary. The main aim of our study was to investigate differences in the architecture of affective lability in different psychosis spectrum disorders, and if putative differences remained when we controlled for current symptom status.

**Methods:**

Affective lability was measured with The Affective Lability Scale Short Form (ALS-SF) in participants with schizophrenia (SZ, n = 76), bipolar I disorder (BD-I, n = 105), bipolar II disorder (BD-II, n = 68) and a mixed psychosis-affective group (MP, n = 48). Multiple analyses of covariance were conducted to compare the ALS-SF total and subdimension scores of the diagnostic groups, correcting for current psychotic, affective and anxiety symptoms, substance use and sex. Double generalized linear models were performed to compare the dispersion of affective lability in the different groups.

**Results:**

Overall group differences in affective lability remained significant after adjusting for covariates (p = .001). BD-II had higher affective lability compared to SZ and BD-I (p = .004), with no significant differences between SZ and BD-I. There were no significant differences in the contributions of ALS-SF dimensions to the total affective lability or in dispersion of affective lability between the groups.

**Conclusions:**

This study provides the construct of affective lability in psychosis spectrum disorders with more granular details that may have implications for research and clinical care. It demonstrates that despite overlap in core symptom profiles, BD-I is more similar to SZ than it is to BD-II concerning affective lability and the BD groups should consequently be studied apart. Further, affective lability appears to be characterized by fluctuations between depressive- and other affective states across different psychosis spectrum disorders, indicating that affective lability may be related to internalizing problems in these disorders. Finally, although the level varies between groups, affective lability is evenly spread and not driven by extremes across psychosis spectrum disorders and should be assessed irrespective of diagnosis.

**Supplementary Information:**

The online version contains supplementary material available at 10.1186/s40345-021-00238-0.

## Background

Affective lability, the propensity to experience rapid, unpredictable and excessive changes in affective states (Zwicker et al. 2019), is a central and common feature of affective instability that is associated with negative outcomes across psychiatric disorders (Patel et al. 2015; Marwaha et al. 2013a, 2014a, 2018; Broome et al. 2015a,b; McDonald et al. 2020). As a consequence, affective lability and other elements of affective instability are gradually becoming recognized as dimensional and transdiagnostic constructs in line with the Research Domain Criteria (RDoC) project of The National Institute of Mental Health (NIMH) (Broome et al. 2015b; Insel et al. 2010; Fernandez et al. 2016).

Due to the considerable overlap in the symptomatology and etiology of schizophrenia and bipolar disorder, the Kraepelinian dichotomy is increasingly being questioned (Craddock and Owen 2010; Pearlson 2015). Consequently, investigating the full spectrum of these disorders—referred to as psychosis spectrum disorders—is recommended when exploring both biomarkers and clinical features (Guloksuz and Os 2018). Still, studies investigating affective lability thus far have focused on bipolar disorder (BD), which is likely to be due to the fluctuations in affective states inherently tied to a BD diagnosis. Affective lability has been found to be both a trait- and a state-dependent factor in individuals with bipolar I (BD-I) and bipolar II (BD-II) disorders. It is present in periods of euthymia (Henry et al. 2008), early in the course of illness (Aminoff et al. 2012), in all polarities of the illness episodes (Henry et al. 2003; Gershon and Eidelman 2015; Faurholt-Jepsen et al. 2015; Verdolini et al. 2019), as well as in non-affected relatives (Hafeman et al. 2016; Birmaher et al. 2013). Due to its associations with adverse clinical correlates such as alcohol use disorders (AUD) (Lagerberg et al. 2017), suicidality (Aas et al. 2017; Ducasse et al. 2017), anxiety disorders (Aas et al. 2017), cardiometabolic risk (Dargel et al. 2018) and inflammation (Dargel et al. 2017), there is mounting evidence that affective lability may be a relevant therapeutic target in BD.

In schizophrenia (SZ), there has previously been limited emphasis on the prevalence and correlates of affective lability, despite indications that it can be a prominent facet of psychotic experiences (Patel et al. 2015; Marwaha et al. 2014b). Indeed, in our recently published study investigating affective lability across psychotic disorders, we found that affective lability was markedly elevated in individuals with SZ and BD compared to healthy controls, with equally high elevations in both groups (Høegh et al. 2020). Also, affective lability was significantly and independently associated with higher levels of positive psychotic- and depressive symptoms in SZ, and with higher levels of AUD and depressive symptoms in BD. This suggests that affective lability adds to the total illness burden across psychosis spectrum disorders. The findings highlight that increased awareness of affective lability in both research and clinical care is warranted.

To further elucidate the mechanisms of affective lability and its potential value as a treatment target consistent with personalized approaches to psychiatry, it is of interest to explore how and if affective lability varies between the specific disorders included in the psychosis spectrum. In line with this, a few previous studies with relatively small samples, as well as two larger studies, have found higher affective lability in individuals with BD-II compared with BD-I (Faurholt-Jepsen et al. 2015, 2019; O'Donnell et al. 2018; Marwaha et al. 2016). Knowledge about the expression of affective lability in SZ and how this expression overlaps with the broader psychosis spectrum, however, is scarce. Also, it is unclear if there are certain types of lability in affect that are more prominent in the different psychosis spectrum disorders. The presence of anxiety, for example, has been found to be high in both individuals with BD and SZ (Karpov et al. 2016; Achim et al. 2011), but to which extent it is part of the composition of affective lability in the various disorders is not known. For potential intervention efforts to be as targeted as possible, more precise knowledge about which affects that appear to be involved in the lability in the different diagnostic subgroups is needed.

Using the same sample as in our previous study (Høegh et al. 2020) together with newly added participants, we are now able to further explore affective lability measured by The Affective Lability Scale Short Form (ALS-SF) across specific psychosis spectrum disorders. These include SZ, BD-I, BD-II in addition to a mixed “psychosis-affective” (MP) group including schizoaffective disorder and psychotic disorders not otherwise specified (psychosis NOS) with prominent mood symptoms. Here, we aim to investigate if these groups exhibit differences in the architecture of affective lability that could aid further characterization of this phenomenon in psychosis spectrum disorders. We also aim to investigate whether putative differences in affective lability between the diagnostic groups are primarily mediated by differences in their current symptomatology. Finally, we explore potential variations in affective lability within the diagnostic categories in the psychosis spectrum, i.e. between individuals with the same type of disorder, by examining the within-group dispersion of ALS-SF scores, and whether this differs between the diagnostic groups. Such an investigation has, to our knowledge, yet to be conducted in this population and will highlight whether affective lability appears to be driven by subgroups with extreme scores or is more evenly spread within and across the different diagnostic groups.

Accordingly, we seek to investigate affective lability in four diagnostic groups within the psychosis spectrum: SZ, BD-I, BD-II and MP, and more specifically to:

1. Investigate (a) if there are differences in the total- and subdimension scores of ALS-SF between the groups, (b) if potential differences remain after controlling for current symptoms, and (c) which of the ALS-SF subdimensions contribute most to the total affective lability score in each of the diagnostic groups.
2. Investigate the dispersion of the ALS-SF scores within each diagnostic category and if there are differences in dispersion between the diagnostic groups.

## Material and methods

### Design

The current study is part of the larger Thematically Organized Psychosis (TOP) study at the Norwegian Center for Mental Disorders Research (NORMENT) in Oslo, Norway. Recruitment to the TOP study has been ongoing since 2003 and potential participants are referred via psychiatric inpatient and outpatient units, including specialized psychosis units as well as community teams, in a catchment area that is comprised of all the major hospitals in Oslo. As such, the overall representability of the sample is considered to be very good. The participants are given thorough information about the voluntary nature of the study and the possibility to withdraw at any time. The participants included in the study have to meet diagnostic criteria for a Diagnostic and Statistical Manual of Mental Disorders 4th Edition (DSM-IV) diagnosis of schizophrenia- or bipolar spectrum disorder and provide informed consent. Both individuals with established diagnoses and individuals who are diagnosed for the first time are included in the study. Further inclusion criteria are intelligence quotient (IQ) above 70, no prior history of severe head trauma and sufficient understanding of a Scandinavian language (Ringen et al. 2008).

### Participants

The sample for the current study was comprised of two hundred and ninety-seven participants with psychosis spectrum disorders from the TOP study, and only participants who had completed the ALS-SF were included. The ALS-SF was originally introduced in a TOP study sub-protocol for participants with first episode mania, and a few years later included in the main protocol (i.e. to patients with other diagnoses than BD-I). It was mainly presented for participants with low levels of current affective symptoms. The diagnostic grouping in the current study was as follows: SZ (including schizophreniform [n = 14]) n = 76, BD-I n = 105, BD-II = 68 and MP (including psychosis NOS [n = 32] and schizoaffective disorder [n = 16]) n = 48. The rationale for combining psychosis NOS and schizoaffective disorder into one “mixed” group was that these categories typically include patients with both psychotic- and affective symptoms that are diagnostically more heterogeneous than the other groups (Santelmann et al. 2015, 2016; Widing et al. 2020). Of the present sample, n = 222 were used in our previous study investigating affective lability across the psychosis spectrum (Høegh et al. 2020), and are now re-analyzed along with the newly added participants to shed light on putative differences in affective lability between the specific psychotic disorders.

### Clinical assessments

The diagnoses in the study were established by the Structured Clinical Interview for DSM-IV axis I disorders (SCID-1), modules A-E (First et al. 1995) which was carried out by trained medical doctors, psychiatrists or clinical psychologists. In the TOP study, diagnostic reliability is assessed with regular intervals and Cohen’s kappa for diagnosis in the range between 0.92 and 0.99 has been found across different assessment teams. To assess current symptom state, the positive subscale of the Positive and Negative Syndrome Scale [PANSS (Kay et al. 1987)] was used for positive psychotic symptoms, and the depression item (G6) and the anxiety item (G2) in the general scale of the PANSS were used for depressive- and anxiety symptoms, respectively. PANSS G6 and PANSS G2 were chosen because they were the only measures of depression and anxiety that were collected at the same time point as the ALS-SF for all participants. The rating for G6 is based on the answer to one initial question (“how has your mood been in the past week, mostly good or mostly bad?”) followed by 1–11 follow-up questions concerning the extent of the depressive state and its behavioral consequences. For G2, the rating is based on the same algorithm; one initial question (“have you been feeling worried or nervous in the past week?”) and then 1 to 6 follow-up questions depending on the response to the first question. The Young Mania Rating Scale [YMRS (Young et al. 1978)] was used to assess manic symptoms. The Alcohol Use Disorders Identification Test [AUDIT (Saunders et al. 1993)] was used to evaluate the degree of harmful alcohol consumption, and drug related problems were measured with the Drug Use Disorders Identification Test [DUDIT (Berman et al. 2005)]. Duration of illness was estimated based on the age of onset of the first SCID-verified episode of psychosis for SZ and MP, and the first SCID-verified affective episode for BD-I and BD-II.

### The Affective Lability Scale Short Form (ALS-SF)

To measure affective lability, we used the ALS-SF (Oliver and Simons 2004). The ALS-SF captures the total level of affective lability reported by an individual, but also subdimensions of affective lability covering oscillations between three subdomains: anxiety-depression, depression-elation and anger and normal mood. Thus, the scale provides an indication of whether affective lability is predominantly driven by specific- or a combination of affects. The ALS-SF has been found to have good psychometric properties (Aas et al. 2015; Look et al. 2010) and is widely used across different clinical populations. The 18 items of the scale are rated on a four-point Likert scale ranging from 0 (“very uncharacteristic of me”) to 3 (“very characteristic of me”) and yields a total score of affective lability as well as scores for the three subdomains. There are no validated cut-off scores for evaluating the severity of affective lability, but in our previous study we found mean ALS-SF total and subscale scores in the range of 0.17–0.39 for healthy controls, 0.85–1.33 for individuals with BD, and 0.69–1.34 for individuals with SZ (Høegh et al. 2020). This corresponds well with what has been found in at least one similar study (Marwaha et al. 2018).

### Statistical analyses

Descriptive statistics, including means with standard deviations or frequencies with percentages where relevant, were used to investigate demographical and clinical characteristics of the different diagnostic groups. The groups were then compared using one-way between-groups analyses of variance (ANOVA) and chi-square tests. The Tukey’s honestly significant difference (HSD) test was used for post-hoc comparisons where appropriate. To investigate group differences in the level of affective lability as measured by scores on the total- and subdimensions of the ALS-SF, a one-way between-groups multivariate analysis of variance (MANOVA) was conducted with Bonferroni post-hoc tests. A multiple analysis of covariance (MANCOVA) was then carried out to investigate if statistically significant group differences in affective lability remained significant when current symptom and substance use status were entered as covariates. The variables which were significantly associated with diagnostic group and/or with the ALS-SF domains in bivariate correlation analyses were entered as covariates. The selected covariates were as follows: current level of positive psychotic symptoms (PANSS P), manic symptoms (YMRS), depression (PANSS G6), anxiety (PANSS G2), alcohol use (AUDIT) and drug use (DUDIT) (see Table 1 for correlation coefficients). In addition to the symptom and substance use variables, sex was entered as a covariate as there was a significant difference in the number of females in the different groups and previous research has indicated that affective lability is higher in females in general (Marwaha et al. 2013b; Winkler et al. 2004). Age, which has also been found to be associated with affective lability previously (Broome et al. 2015b), was not associated with the diagnostic groups or with the ALS-SF scores in our sample and therefore not included in further analyses. Effect sizes were calculated by partial eta squared. To investigate which of the ALS-SF subdimensions contributed the most to the total affective lability, a one-way repeated measures ANOVA was performed for each group. The Statistical Package for the Social Sciences (SPSS Inc., Chicago, IL, version 26) was used for all statistical analyses and a significance level of p ≤ 0.05 (two-tailed tests) was employed. For aim 2, ALS-SF scores for all dimensions for each diagnostic group were plotted into the Graphpad Prism tool (GraphPad Software, La Jolla California USA, version 8.0 for Windows) and converted into violin plots to illustrate score distributions. To further investigate variability in the ALS-SF scores, double generalized linear models (DGLM, Y ~ Dx, ~ Dx) were carried out using R (R core team 2017) to test if the ALS-SF score dispersions were significantly different between groups (for more detailed information see Additional file 1).

**Table 1:** Bivariate correlation analyses

	PANSS P	PANSS G2	PANSS G6	AUDIT	DUDIT	YMRS	SEX
ALS-SF total	r_s_ = 0.174**	r_s_ = 0.381**	r_s_ = 0.305**	r_s_ = 0.153**	r_s_ = 0.227**	r_s_ = 0.282**	r_s_ = 0.136*

*ALS-SF* affective lability scale short form, *PANSS P* positive and negative syndrome scale positive subscale, *PANSS G2 *positive and negative syndrome scale anxiety item, *PANSS G6* positive and negative syndrome scale depression item, *AUDIT* the alcohol use disorders identification test, *DUDIT* the drug use disorders identification test, *YMRS* young mania rating scale. *p < 0.05, **p < 0*.001*

## Results

### Demographics and clinical characteristics of the sample

Demographic and clinical characteristics for SZ, BD-I, BD-II and MP are presented in Table 2. A statistically significant difference in sex between the groups was found, with fewer women in the SZ group compared to remaining groups. There were also significantly more women in the BD-II compared to the BD-I group. In addition, the duration of illness between the groups was significantly different; the BD-I and BD-II groups had been ill longer than the SZ and MP groups (p < 0.001 and p < 0.05, respectively). Regarding clinical features, there were statistically significant differences between the groups for PANSS total, PANSS P, YMRS, G2 anxiety and AUDIT (see Table 2)*.*

**Table 2:** Demographics and clinical characteristics

	SZ* (n* = *76)*	BD-I *(n* = *105)*	BD-II *(n* = *68)*	MP* (n* = *48)*		Statistics		*P*-value
	Mean (SD)	Mean (SD)	Mean (SD)	Mean (SD)				
Age, years	29.8 (9.2)	31.6 (11.2)	29.4 (9.2)	29.5 (8.3)		F = 0.971, df = 3		0.407
Female sex, n (%)	27 (35.5)	56 (53.3)	47 (69.1)	27 (56.3)		X^2^ = 16.608, df = 3		*0.001* SZ < all, BD-II > BD-1
Education, years	14.3 (3.1)	15.0 (2.9)	16.0 (2.8)	14.6 (3.1)		F = 1.950, df = 3		0.122
Duration of illness, years	4.1 (6.2)	9.9 (9.8)	13.1 (8.7)	6.1 (7.2)		F = 15.785, df = 3		*0.000* BD-I and BD-II > SZ, MP
PANSS—total	56.2 (15.6)	42.1(8.4)	43.0 (8.2)	53.8 (14.2)		F = 30.356, df = 3		*0.000* SZ, MP > BD-I, BD-II
PANSS—P	12.8 (4.8)	9.0 (2.5)	9.1 (2.6)	12.1 (3.8)		F = 28.431, df = 3		*0.000* SZ, MP > BD-I, BD-II
Depression (PANSS item G6)	2.3 (1.2)	2.3 (1.4)	2.7 (1.4)	2.4 (1.3)		F = 2.167, df = 3		0.092
Anxiety (PANSS item G2)	2.8 (1.2)	2.7 (1.3)	3.3 (1.4)	2.5 (1.2)		F = 3.599, df = 3		*0.014* BD-II > BD-I, MP
YMRS—total	3.5 (4.2)	2.3 (3.4)	3.0 (4.2)	1.7 (2.3)		F = 2.699, df = 3		0.046*
AUDIT total	5.1 (4.9)	8.1 (7.1)	8.5 (6.4)	5.3 (5.1)		F = 5.648, df = 3		*0.001* SZ < BD-I, BD-II; MP < BD-II
*DUDIT total*	*3.1 (6.9)*	*3.6 (7.1)*	*2.8 (5.6)*	*3.2 (7.1)*		*F* = 0*.237, df* = *3*		*0.871*

Statistically significant group differences are highlighted in italic

*SZ* schizophrenia, *BD* bipolar disorder, *MP* mixed psychosis, *PANSS* positive and negative syndrome scale, *YMRS* young mania rating scale, *AUDIT* the alcohol use disorders identification test, *DUDIT* drug use disorders identification test

*Post-hoc tests were non-significant

### Affective lability in the diagnostic groups: total and subdimension scores

**Fig. 1:**
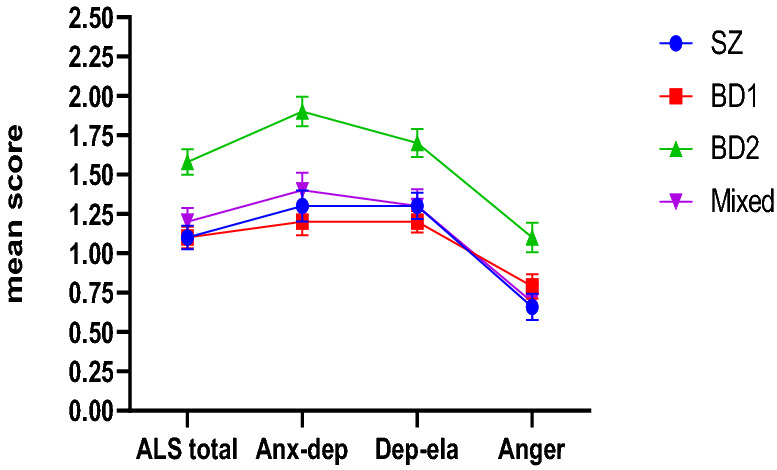
ALS-SF mean and SEM scores. *SEM* standard error of the mean, *SZ* schizophrenia, *BD* bipolar disorder, *ALS-SF* affective lability scale short form, *anx-dep* anxiety-depression domain, *dep-ela* depression-elation domain

The mean scores for the ALS-SF dimensions for the different diagnostic groups are presented in Fig. 1. The MANOVA showed that there was a statistically significant difference in affective lability between the groups for all of the ALS dimensions: ALS-total F = 8.446, df = 3, p < 0.001; ALS anxiety-depression F = 9.298, df = 3, p < 0.001; ALS depression-elation F = 7.281, p < 0.001 and ALS anger F = 4.252, p = 0.006. Effect sizes were moderate; partial eta^2^ = 0.080, 0.087, 0.069 and 0.042, respectively.

Post-hoc analyses with Bonferroni revealed that the BD-II group had significantly higher affective lability scores compared to all of the other groups for the ALS total (p < 0.05) and depression-elation (p < 0.05) dimensions. For the ALS anxiety-depression dimension, the scores for the BD-II group were significantly higher than those of the BD-I and SZ groups (p < 0.001 for both), but not the MP group (p = 0.060). Finally, the BD-II group had significantly higher scores compared to the SZ group (p = 0.006) and the MP group (p = 0.042) groups, but not the BD-I group (p = 0.089) on the anger dimension. There were no significant differences in the scores for the SZ and BD-I groups on any dimension (p = 1.000).

### Results from multivariate analyses

The overall differences in affective lability between the groups remained statistically significant also after adjusting for the effects of sex, current symptom- and substance use status: ALS total F = 5.305, df = 3, p = 0.001; ALS anxiety-depression F = 6.139, df = 3, p < 001; ALS depression-elation F = 4.432, df = 3, p = 0.005 and ALS anger F = 4.184, df = 3, p = 0.006. Again, the effect sizes were moderate with partial eta^2^ of 0.057, 0.067, 0.048 and 0.046. Post-hoc group comparisons with Bonferroni showed that the difference in AL between BD-II versus SZ and BD-I remained statistically significant for the ALS total (p = 0.004 for both SZ and BD-I), anxiety-depression (SZ p = 0.038, BD-I p = 0.001) and depression-elation dimensions (SZ p = 0.031, BD-I p = 0.006). The difference between BD-II and SZ on the anger domain also remained statistically significant (p = 0.004). The difference between the MP group and the BD-II group no longer remained statistically significant for the total-, depression-elation- and anger domains after adjusting for covariates.

### Contributions of the ALS-SF subdimensions to the total score

In all of the diagnostic groups, the one-way repeated measures ANOVAs with Bonferroni post-hoc tests showed that the anxiety-depression and depression-elation dimensions contributed most to the total affective lability, with no significant differences in the level of scores between these dimensions in any group (p = 1.000 for SZ and BD-I, p = 0.225 for BD-II, p = 0.814 for MP). Across the board, the anger dimension was found to contribute significantly less to the total score compared to the anxiety-depression and depression-elation dimensions (p < 0.001).

### Dispersion of affective lability scores within and between the diagnostic groups

**Fig. 2:**
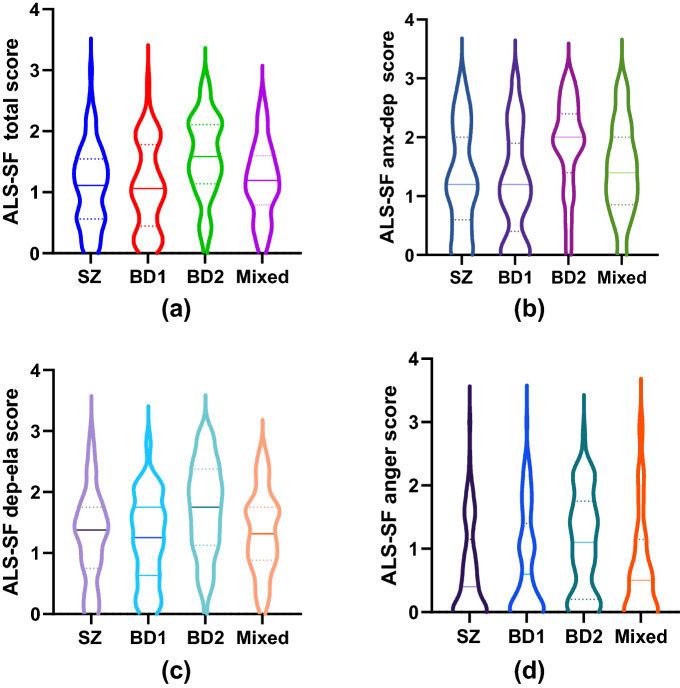
**a**–**d** ALS-SF score distribution: **a** ALS-SF total score, **b** anxiety-depression dimension, **c** depression-elation dimension, **d** anger dimension. *SZ* schizophrenia, *BD* bipolar disorder, *ALS-SF* affective lability scale short form, *anx-dep* anxiety-depression dimension, *dep-ela* depression-elation domain

The score distributions for all dimensions of the ALS-SF for the four diagnostic groups are shown in Fig. 2a–d. The median score is illustrated by a vertical straight line. The double generalized linear models found no significant differences (p > 0.05) in the score dispersions between any of the groups in any of the domains, indicating that despite differences in mean ALS-SF scores between groups, the dispersion in the scores was the same (for more information see Additional file 1).

## Discussion

The construct of affective lability refers to the propensity to experience rapid, unpredictable and excessive changes in affective states (Zwicker et al. 2019). The main aim of the current study was to investigate differences in affective lability between different psychosis spectrum disorders. Our results show that individuals with BD-II had markedly elevated levels of total- and subdimension affective lability compared to BD-I and SZ, even when correcting for sex, the level of current symptoms and substance use. Affective lability in the MP group, on the other hand, was not significantly different from BD-II nor SZ or BD-I when the covariates were taken into account. There were no statistically significant differences between individuals with BD-I and SZ for any ALS-SF dimension and these two groups had very similar score patterns throughout. This suggests that despite the overlap in core affective symptom profiles of BD-I and BD-II, the BD-I group is more similar to SZ than it is to BD-II concerning levels of affective lability. Further, since we controlled for current symptoms, our findings imply that there are some trait-like differences between the disorders with respect to affective lability, where elevated affective lability is perhaps more inherently tied to BD-II compared to the other psychosis spectrum disorders.

Given that individuals with BD-II are more similar to healthy controls when it comes to neurobiology, genetics and cognition compared to BD-I and SZ (MacQueen et al. 2005), one can speculate if the high affective lability is based in environmental or clinical risk factors specific to BD-II. For instance, childhood trauma has been found to be associated with affective lability (Aas et al. 2014), but the prevalence rates of trauma are reported to be at the same level in individuals with BD-I and BD-II (Palmier-Claus et al. 2016; Janiri et al. 2015). The presence of comorbid Attention-Deficit/Hyperactivity Disorder (ADHD) and anxiety disorders is also likely to increase affective lability (Broome et al. 2015a; Aas et al. 2017), but again the rates are at the same level in BD-I versus BD-II (Pataki and Carlson 2013; Bennett et al. 2019; Brus et al. 2014; Pavlova et al. 2015). It is perhaps more plausible that the elevation in affective lability observed in individuals with BD-II is associated with more frequent and severe borderline personality traits (Saunders et al. 2020) and/or higher rates of depressive episodes and symptoms (Karanti et al. 2020), but this needs to be investigated further. In addition, affective lability, anxiety disorders, borderline personality disorders and BD-II appear to be more prevalent in women compared to men, and there may be some interrelationships here that are worthwhile looking into in future research. Regardless of origin, it seems important to investigate the BD groups separately with regards to affective lability. When it comes to the similarities between individuals with BD-I and SZ, this may not be surprising at least from a genetic point of view, given the established overlap between BD-I and SZ (Tamminga et al. 2013; Tesli et al. 2014). It is, however, important to keep in mind that both individuals with BD-I and SZ also present with higher affective lability rates than healthy controls (Høegh et al. 2020).

With respect to the architecture of affective lability, oscillations between anxiety-depression and depression-elation were the most typical for all groups. In our previous study, we found significant associations between affective lability and current depression in both SZ and BDs (Høegh et al. 2020). We now extend these findings to show that even when controlling for the level of depressive symptoms, significant between-group differences in affective lability persist. Somewhat surprisingly, we found that fluctuations between depression and elation were also prevalent, even in individuals with SZ, along with fluctuations involving anxiety. This is in line with the dimensional perspective of psychosis that has been found in previous research showing a large degree of symptomatic overlap between disorders (Os and Kapur 2009). The mean scores of the SZ, BD-I and MP groups on the anger dimension were low (0.66, 0.79 and 0.69, respectively). Although the anger score for the BD-II group was higher (1.08), it is still comparatively low relative to the BD-II scores for the other dimensions. Collectively, this is an indication that affective lability is related to more internalizing versus externalizing problems and behaviors in psychotic disorders, which is different from the pattern found in for example borderline personality disorder where rapid shifts involving anger appear to be more characteristic (Henry et al. 2001; Koenigsberg et al. 2002).

To our knowledge, the dispersion of ALS-SF scores in the different psychosis spectrum disorders has not previously been investigated. The violin plots showing the full distributions of the data confirm that the scores seem to be relatively evenly dispersed in all groups, i.e. the scores are not clustered around the minimum or maximum but rather around the median score. Hence, the level of affective lability in the groups does not appear to result primarily from the presence of subgroups of patients with extreme scores, but to represent the typical score pattern for each disorder. Visually, the shape of the distribution of scores in the BD-II group stands out, especially for the anxiety-depression dimension. Yet, the statistical analyses revealed no significant differences between the groups with regards to dispersion. This indicates that although the level varies between groups, affective lability is evenly spread and not driven by extremes across psychosis spectrum disorders and should be routinely assessed irrespective of diagnosis.

### Limitations and strengths

The present study must be interpreted in light of some limitations. The cross-sectional nature of the study implies that we cannot make causal attributions about the association between diagnostic group and affective lability. In addition, data on comorbid anxiety disorders, personality disorders and ADHD are lacking, and the measures used for anxiety and depressive symptoms are based on a scale primarily developed for assessing psychotic symptoms. Hence, the possibility that current symptoms still could have influenced the association between affective lability and diagnostic group cannot be ruled out completely. However, we believe that the likelihood of this is limited due to the relatively low levels of anxiety and depressive symptoms. Further, the risk of recall- and response bias cannot be ruled out as the ALS-SF is a self-report instrument. The study also has several strengths; it has a large, diagnostically well-characterized sample covering the psychosis spectrum, and uses a multidimensional assessment scale of affective lability that provides a richer insight and understanding of the construct.

### Conclusions

Our results illustrate that affective lability is more prominent in individuals with BD-II compared to SZ and BD-I, and that this is not explained by differences between the groups in sex, levels of affective-, psychotic- or anxiety symptoms or severity of substance use. BD-II thus appears to be a particularly vulnerable diagnostic group with respect to affective lability. No differences in affective lability were found between individuals with BD-I and SZ. The results further add information about the structure of affective lability in these disorders emphasizing the significance of fluctuations between depressive- and other affective states. The findings also show that there is an even dispersion of affective lability scores within each diagnostic group, and that the dispersion also appears to be largely equivalent across groups. Overall, the study provides the concept of affective lability in psychotic disorders with more granularity by showing differences and similarities between diagnostic groups that may have implications for both research and clinical practice.

## Supplementary Information

**Additional file 1: **Double Generalized Linear Models.

## Data Availability

The data that support the findings of this study will be made available upon reasonable request.

## Abbreviations

ALS-SF: Affective Lability Scale Short Form
SZ: Schizophrenia
BD: Bipolar disorder
RDoC: Research Domain Criteria
NIMH: The National Institute of Mental Health
AUD: Alcohol Use Disorder
MP: Mixed Psychosis group
Psychosis NOS: Psychosis Not Otherwise Specified
TOP: Thematically Organized Psychosis Study
NORMENT: Norwegian Center for Mental Disorders Research
DSM-IV: Diagnostic and Statistical Manual of Mental Disorders 4th Edition
SCID: Structured Clinical Interview for DSM-IV
PANSS: Positive and Negative Syndrome Scale
YMRS: Young Mania Rating Scale
AUDIT: Alcohol Use Disorders Identification Test
DUDIT: Drug Use Disorders Identification Test
ANOVA: Analysis of Variance
HSD: Honestly Significant Difference test
MANOVA: Multivariate Analysis of Variance
MANCOVA: Multivariate Analysis of Covariance
SPSS: Statistical Package for the Social Sciences
DGLM: Double Generalized Linear Models
ADHD: Attention-Deficit/Hyperactivity Disorder
