# Supplementary material for: Characterization of affective lability across subgroups of psychosis spectrum disorders
Source: Int J Bipolar Disord. 2021 Nov 4;9:34. doi: 10.1186/s40345-021-00238-0 (PMC8566621; doi:10.1186/s40345-021-00238-0)
Supplement: Supplementary file 1 — Additional file 1: Double Generalized Linear Models. [file 40345_2021_238_MOESM1_ESM.docx]

**Additional file 1: Double Generalized Linear Models**

Double Generalized Linear Models method:

We estimated mean and dispersion parameters across the four variables, where Y is the mean in the first GLM, and the dispersion of the first GLM are the responses in the second. We then obtain an estimate of the difference in dispersion around the mean between SZ/BDI, SZ/BDII, SZ/MP, BDI/BDII, BDI/MP, and BDII/MP for all the variables.

Results:

#### DGLM - SZ_BD1 ####

variable t Dx p Dx t-disp Dx p-disp Dx

1 ALStotal -0.1086375 0.9136117 0.36642226 0.7144828

2 ALS.anx_dep -0.6690282 0.5043394 0.08162557 0.9350357

3 ALS.dep_ela -0.4853188 0.6280437 -0.15551411 0.8765913

4 ALSanger 1.1623204 0.2466527 0.67678609 0.4994153

#### DGLM - SZ_BD2 ####

variable t Dx p Dx t-disp Dx p-disp Dx

1 ALStotal 4.265360 3.623396e-05 0.006194024 0.9950666

2 ALS.anx_dep 4.169718 5.280471e-05 -0.738180938 0.4616227

3 ALS.dep_ela 3.599835 4.391202e-04 0.038836585 0.9690752

4 ALSanger 3.377789 9.431697e-04 0.635133058 0.5263637

#### DGLM - SZ_mixed ####

variable t Dx p Dx t-disp Dx p-disp Dx

1 ALS.total 0.7088941 0.4797427 -0.3953241 0.6932941

2 ALS.anx_dep 1.0850939 0.2800196 -0.7993194 0.4256587

3 ALS.dep_ela 0.5118553 0.6096776 -0.7790640 0.4374506

4 ALS.anger 0.2185988 0.8273278 0.5945664 0.5532344

#### DGLM - BD1_BD2 ####

variable t Dx p Dx t-disp Dx p-disp Dx

1 ALS.total 4.565237 9.501806e-06 -0.40446791 0.6863741

2 ALS.anx_dep 5.179133 6.213769e-07 -0.64118808 0.5222589

3 ALS.dep_ela 4.361734 2.224544e-05 0.18509421 0.8533745

4 ALSanger 2.413508 1.685580e-02 -0.05335712 0.9575097

#### DGLM - BD1_mixed ####

variable t Dx p Dx t-disp Dx p-disp Dx

1 ALS.total 0.8363693 0.40426879 -0.83461614 0.4052524

2 ALS.anx_dep 1.7819800 0.07676224 -0.61621247 0.5386821

3 ALS.dep_ela 1.0038530 0.31705540 -0.64213553 0.5217589

4 ALS.anger -0.7257179 0.46913502 0.06288461 0.9499416

#### DGLM - BD2_mixed ####

variable t Dx p Dx t-disp Dx p-disp Dx

1 ALS.total -3.266640 0.001438062 -0.4970330 0.6201230

2 ALS.anx_dep -2.813284 0.005776867 -0.0486473 0.9612855

3 ALS.dep_ela -2.901368 0.004460089 -0.7506953 0.4543828

4 ALS.anger -2.644985 0.009321782 0.1094278 0.9130556
